# The reproductive ecology drivers of egg attendance in amphibians

**DOI:** 10.1111/ele.14109

**Published:** 2022-10-01

**Authors:** Andrew I. Furness, Isabella Capellini

**Affiliations:** ^1^ Department of Biological and Marine Sciences University of Hull Hull UK; ^2^ Energy and Environment Institute, University of Hull Hull UK; ^3^ School of Biological Sciences Queen's University Belfast Belfast UK

**Keywords:** amphibians, egg attendance, parental care, phylogeny, reproductive ecology

## Abstract

Parental care is extremely diverse but, despite much research, why parental care evolves is poorly understood. Here we address this outstanding question using egg attendance, the simplest and most common care form in many taxa. We demonstrate that, in amphibians, terrestrial egg deposition, laying eggs in hidden locations and direct development promote the evolution of female egg attendance. Male egg attendance follows the evolution of hidden eggs and is associated with terrestrial egg deposition but not with direct development. We conclude that egg attendance, particularly by females, evolves following changes in reproductive ecology that are likely to increase egg survival, select for small clutches of large eggs and/or expose eggs to new environmental challenges. While our results resolve a long‐standing question on whether reproductive ecology traits are drivers, consequences or alternative solutions to caring, they also unravel important, yet previously unappreciated, differences between the sexes.

## Graphical abstract

Parental care is extremely diverse but, despite much research, why parental care evolves is poorly understood. Here we address this outstanding question using egg attendance, the simplest and most common care form in many taxa. Our results suggest that amphibian egg attendance, particularly by females, evolves following changes in reproductive ecology that are likely to increase egg survival, select for small clutches of large eggs, and/or expose eggs to new environmental challenges.

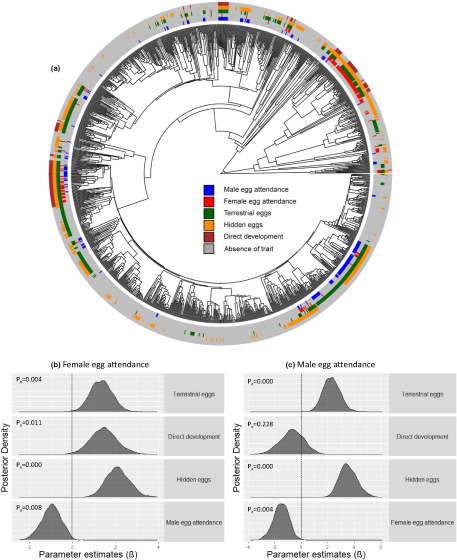

## INTRODUCTION

While parents in many species do not provide care for their offspring after fertilisation, parental care has evolved numerous times and diversified into many forms across the animal kingdom (Smiseth et al., 2012). Theoretical and empirical studies demonstrate that parental care influences the fitness of parents and offspring, evolves together with life‐history traits, promotes the evolution of sociality and plays a central role in sexual conflict, parent–offspring conflict and sibling competition (Alonzo, 2010; Furness et al., 2022; Halliwell et al., 2017; Houston et al., 2005; Parker et al., 2002; West & Capellini, 2016). Surprisingly, which ecological drivers favour the initial evolution of parental care remains unclear. Answering this question is important given the wide‐ranging implications of parental care but it is challenging because few species exhibit genetically determined differences between populations in parental care or plasticity in the expression of care; moreover, behavioural responses over ecological time scales may not reflect the selective pressures responsible for the origin of traits (Kokko & Jennions, 2012). Finally, previous comparative studies find correlations between parental care and ecological conditions but these cannot reveal whether reproductive ecology is the driver or consequence of the evolution of parental care. A powerful approach to address the question of the origin of parental care is to exploit groups with high interspecific diversity and adopt a comparative perspective. Here we investigate whether reproductive ecology traits that likely facilitate an initial association between parents and offspring promote the evolutionary origin of egg attendance in amphibians using state‐of‐the‐art phylogenetic comparative methods. We also investigate counter‐hypotheses that propose reverse causation or view egg attendance and reproductive ecology adaptations as alternative solutions to the same challenges.

**FIGURE 1:**
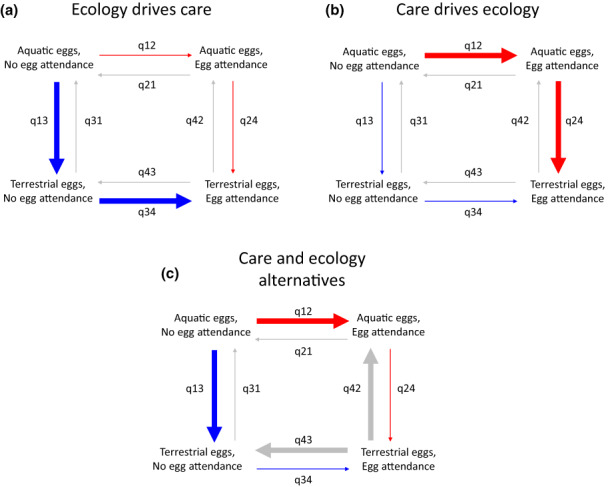
Schematic of predictions and interpretation of BayesTraits discrete‐dependent models for the correlated evolution of egg attendance and reproductive ecology. If the dependent model is the better fitting model (Supplementary Methods), we examine the posterior distributions of its eight transition rates between the four possible combinations of character states to identify evolutionary pathways supporting the predictions of the alternative hypotheses. As an example, here we illustrate the case of aquatic/terrestrial eggs and presence/absence of egg attendance. The arrows indicate the direction of change and their thickness the magnitude of the associated transition rates (the q_ij_) between the four states (aquatic eggs and no egg attendance, aquatic eggs and egg attendance, terrestrial eggs and no egg attendance, terrestrial eggs and egg attendance). (a) Reproductive ecology drives the origin care (blue arrows): If the presence of terrestrial eggs promotes the evolution of egg attendance, the transition rate for the gain of egg attendance is higher with terrestrial eggs than aquatic eggs (q_34_ > q_12_) and/or terrestrial eggs are gained more rapidly from the absence of both traits than egg attendance (q_13_ > q_12_) and this is followed by a rapid gain of egg attendance (q_34_ > 0). (b) Care drives changes in reproductive ecology (red arrows); if egg attendance promotes the acquisition of terrestrial eggs, the transition rate for the gain of terrestrial eggs is faster with egg attendance than without it (q_24_ > q_13_) and/or egg attendance evolves faster than terrestrial eggs from the absence of both traits (q_12_ > q_13_) and promotes the rapid gain of terrestrial eggs (q_24_ > 0). (c) Care and reproductive ecology adaptations are alternatives (red, blue and grey arrows); the combination of terrestrial eggs and egg attendance is lost more rapidly than it is gained (q_43_ > q_34_; q_42_ > q_24_) indicating that it is evolutionarily unstable, while terrestrial eggs without attendance and/or attendance of aquatic eggs are evolutionary stable (q_12_ > q_21_ and q_42_ > q_24_; q_13_ > q_31_ and q_43_ > q_34_). See also Table 1 for further details.

Egg attendance is ideal for unravelling how ecology promotes the origin of parental care. Attendance is the most common (Blumer, 1982; Crump, 1995; Trumbo, 2012) and one of the simplest care behaviours (Furness & Capellini, 2019; Wells, 2007). Specifically, it is expected to evolve easily since it only requires the parent(s) to remain with the offspring at a fixed location (Smiseth et al., 2012). Consistent with this hypothesis, egg attendance in amphibians evolves more quickly than complex care forms (Furness & Capellini, 2019). In support of theoretical predictions that increased parental investment is accompanied by higher offspring survival, attendance increases egg survivorship in numerous taxa (birds: (Andersson & Waldeck, 2006), fishes: (Klug et al., 2005), reptiles: (Pike et al., 2016), amphibians: (Croshaw & Scott, 2005; Delia et al., 2017; Delia et al., 2020; Ospina‐L et al., 2020; Poo & Bickford, 2013; Taigen et al., 1984), arachnids: (García‐Hernández & Machado, 2017) and crustaceans: (Palaoro & Thiel, 2020)). Attending parents protect the offspring against pathogens (Boos et al., 2014; Green, 1999) and predators (Croshaw & Scott, 2005; Delia et al., 2017; García‐Hernández & Machado, 2017; Gibson & Buley, 2004; Kushlan & Kushlan, 1980; Pike et al., 2016), prevent the desiccation of terrestrial eggs (Croshaw & Scott, 2005; Delia et al., 2020; Poo & Bickford, 2013; Taigen et al., 1984), increase aeration of aquatic eggs in low oxygen environments (Blumer, 1982; Karino & Arai, 2006; Salthe & Mecham, 1974; Takahashi et al., 2017), decrease the likelihood of developmental abnormalities through constant manipulation of eggs (Crump, 1995; Lehtinen & Nussbaum, 2003; McDiarmid, 1978; Simon, 1983) and assist terrestrial hatchlings in exiting the nest (Crump, 1995; Merchant et al., 2018). Egg attendance may also trigger the evolution of further parental investment, including care at the tadpole and juvenile stage (Furness & Capellini, 2019) and larger eggs (Furness et al., 2022). Amphibians are well‐suited to study the evolutionary origin of this care behaviour, given that male and female egg attendance have evolved repeatedly (Furness & Capellini, 2019) and their reproductive ecology is diverse. Exploiting such diversity, we investigate three hypotheses and their counter‐hypotheses on how reproductive ecology (terrestrial egg laying, hiding the eggs, direct development) and egg attendance evolved together (Figure 1, Table 1).

**TABLE 1:** Predictions for alternative hypotheses on the correlated evolution of reproductive ecology (terrestrial eggs, hidden eggs and direct development) and parental care (egg attendance)

Hypothesis	Probit model: Direction of the association	Discrete: Supported model	Dependent: Transition rates magnitude
Reproductive ecology drives care	+	Dependent	Care evolves faster when the ecological trait is present: q_34_ > q_12_ and/or ecological trait evolves first: q_13_ > q_12_ and q_34_ > 0
Care drives change in reproductive ecology	+	Dependent	Change in ecology evolves faster when care is present: q_24_ > q_13_ and/or care evolves first: q_12_ > q_13_ and q_24_ > 0
Care and ecology are alternative solutions	−	Dependent	Care alone stable: q_12_ > q_21_ and q_42_ > q_24_ Ecology alone stable: q_13_ > q_31_ and q_43_ > q_34_ Both present unstable: q_43_ > q_34_ and q_42_ > q_24_
Care and reproductive ecology are unrelated	NS	Independent	N/A

*Note*: For each hypothesis we report: The direction of significant predicted effects in the probit model with all variables (positive effects indicated by a plus, negative by a minus); whether in the discrete analysis the dependent or the independent model is best fitting; and, if the dependent model is better fitting, the expected differences in the magnitude of the transition rates between the combination of character states (q_ij_). The predictions using the discrete model framework are illustrated in Figure 1

A classic hypothesis proposes that laying eggs in terrestrial habitats increases egg survivorship compared to aquatic habitats, but the new environmental challenges faced by terrestrial eggs promote the evolution of parental care (Crump, 1995; McDiarmid, 1978; Wells, 2007). Eggs and larvae can suffer high mortality in aquatic environments due to predation by many species (Magnusson & Hero, 1991; Martin & Carter, 2013; Touchon & Worley, 2015; Wells, 2007) including cannibalistic conspecifics (Polis & Myers, 1985). Terrestrial egg deposition can reduce predation risk (Magnusson & Hero, 1991; Touchon & Worley, 2015) and is believed to be a major innovation that increased egg survival since early in tetrapod evolution (Gomez‐Mestre et al., 2012; Vági et al., 2020). Furthermore, terrestrial egg deposition can benefit montane species, whose clutches can be swept away in streams with highly variable currents (Crump, 2015), and terrestrial habitats offer higher oxygen availability to species for which oviposition sites are otherwise poorly oxygenated waters (Wells, 2007). Terrestrial egg development, however, entails new challenges, including risk of desiccation, new pathogens and predators. Although amphibian terrestrial eggs may exhibit adaptations that limit dehydration, such as foam nests or thick jelly layers (Seymour, 1999; Wells, 2007), caring parents can protect against this new mortality risk (Delia et al., 2020; Poo & Bickford, 2013; Taigen et al., 1984), as well as pathogens (Green, 1999) and terrestrial predators (Delia et al., 2017). Terrestrial eggs may also promote the origin of parental care because they are larger and laid in smaller clutches (Furness et al., 2022) and theoretical models predict that larger offspring should favour the evolution of further parental investment, including care (Nussbaum & Schultz, 1989; Trivers, 1972). Because of these reasons, terrestrial egg deposition is expected to evolve first and promote the origin of egg attendance (Table 1, Figure 1a).

Similarly, egg attendance may evolve when eggs are placed in hidden or protected locations that likely increase egg survival, such as under rocks or in holes, because exposed eggs are easier for predators to find (McDiarmid, 1978; Wells, 2007). Thus, hiding eggs has probably evolved to improve egg survival in aquatic and terrestrial habitats (McDiarmid, 1978). However, placing eggs in protected sites should also reduce predation risk for parents and enable them to provide other benefits that enhance egg survivorship, such as prevention of desiccation for terrestrial eggs, increasing oxygenation for aquatic eggs and protection against pathogens. Because amphibian caring parents are susceptible to predation by many species (Delia et al., 2017), hiding the eggs could lower the cost of attendance by decreasing parental mortality risk and increase the benefits of caring by improving egg survival, making the evolution of parental care advantageous. This hypothesis thus predicts that hiding the eggs evolves first and facilitates the evolution of egg attendance (Table 1, Figure 1a).

Finally, we propose that direct development may select for egg attendance. Direct development refers to eggs forgoing the larval stage and hatching as juveniles. Most amphibian eggs with direct development are terrestrial (Callery et al., 2001; Gomez‐Mestre et al., 2012), larger and laid in smaller clutches than those hatching into free‐living tadpoles (Furness et al., 2022). Because larger eggs have prolonged incubation periods, which expose them to predators and other sources of mortality for longer (Wells, 2007), direct‐developing eggs should face increased mortality risk. Thus, selection should favour the evolution of parental care because few, large, direct‐developing eggs are of high reproductive value to the parents (Trivers, 1972) and when the duration of the vulnerable egg stage is long (Nussbaum & Schultz, 1989; Royle et al., 2012). Therefore, direct development should precede the evolution of egg attendance (Table 1, Figure 1a).

Although the origin of parental care has been much discussed (Royle et al., 2012, 2016; Shine, 1978; Trumbo, 2012; Wilson, 1975; Wong et al., 2013), to our knowledge no study to date has formally tested hypotheses on the reproductive ecology drivers of care in amphibians or other taxa. Importantly, alternative hypotheses propose that parental care is the cause—not the consequence—of evolutionary changes in reproductive ecology (Table 1, Figure 1b). Specifically, egg attendance may evolve in aquatic habitats to protect against predation or to improve oxygenation and facilitate the transition to terrestrial egg development (McDiarmid, 1978; Wells, 2007). Likewise, predation on parents attending exposed eggs (Crump, 1995; Delia et al., 2017) may lead to selection for hiding the clutch in secluded sites. Alternatively, hiding the eggs and caring may represent alternative solutions if both are equally effective at increasing egg survival (Table 1, Figure 1c). Finally, egg attendance may evolve first and favour longer time at the egg stage under parental protection (Shine, 1978), and so promote the evolution of direct development. These alternative hypotheses make opposite predictions on causation, hence the order of evolutionary events, than those proposing that changes in reproductive ecology facilitate the evolution of parental care (Figure 1, Table 1).

Uniparental male and female egg attendance have evolved in different regions of the amphibian phylogeny and facilitated the evolution of attendance by the other sex, although biparental egg attendance is evolutionarily unstable leading to the loss of care by one sex (Furness & Capellini, 2019). This raises the question of whether the selective forces promoting the origin of egg attendance differ between the sexes. Hypotheses and counter‐hypotheses on the relationship between reproductive ecology and parental care have however been proposed regardless of which sex cares. Nonetheless, female attendance could be favoured if females lay small clutches of large eggs, such as in species with terrestrial and direct‐developing eggs, given that, unlike for males, female fitness is limited by fecundity and larger eggs represent high fitness return (Smith & Fretwell, 1974; Trivers, 1972).

Here, we investigate whether the evolution of terrestrial eggs, hidden eggs and direct development are drivers or consequences of the evolution of egg attendance in males and females, or whether egg attendance and reproductive ecology traits are alternative adaptations. We employ state‐of‐the‐art phylogenetic comparative methods that can test predictions on the nature of association between parental care and reproductive ecology and allow us to discriminate between alternative hypotheses (Figure 1, Table 1). Specifically, by identifying the order of evolutionary events across the phylogeny (i.e. whether care evolves prior or after a change in reproductive ecology), these methods bring us closer to identifying causation (Pagel, 1994; Pagel & Meade, 2006) than correlational approaches.

## MATERIAL AND METHODS

### Data set

**FIGURE 2:**
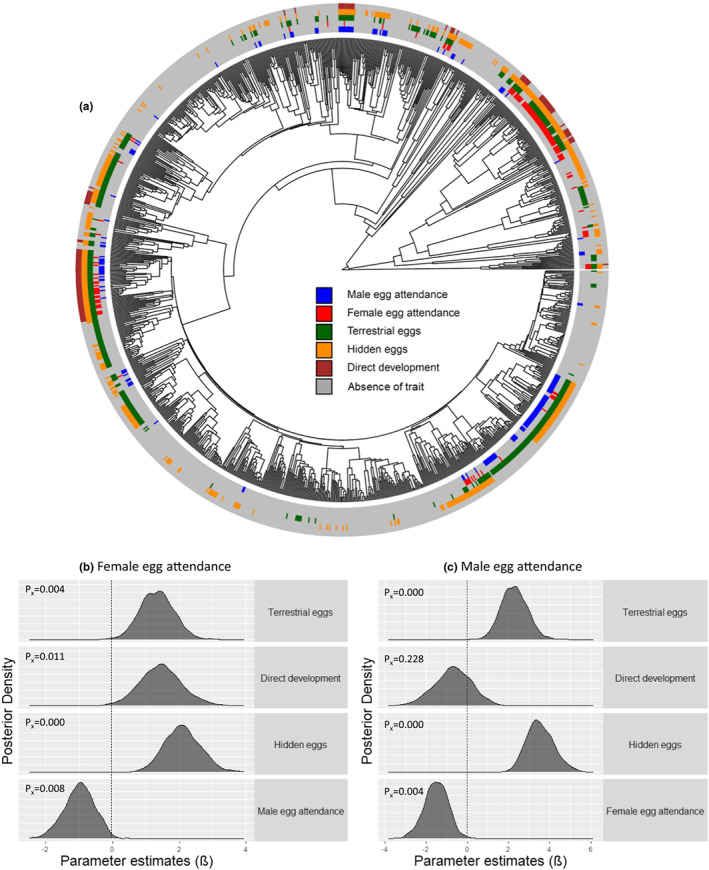
(a) Distribution of the data on the phylogeny in amphibians. From the inner circle to the external circle: Male egg attendance, female egg attendance, terrestrial eggs, hidden eggs and direct development (*n* = 1202 species for all traits). (b, c) posterior density distributions of the parameter estimates from the probit models for the association between sex‐specific egg attendance and reproductive ecology predictors. The posterior distributions of the parameter estimates (β) of the predictors (i.e. terrestrial eggs, direct development, hidden eggs and egg attendance by the opposite sex) are shifted to the right of 0 (dotted vertical line) with positive effects, and left with negative effects. We report the percentage of the posterior distribution that crosses 0 (P_x_) and consider evidence of significance P_x_ < 0.05. Full model details can be found in table S2.

We compiled a data set on presence or absence of the following, binary traits: egg attendance by males and/or females, direct development, terrestrial eggs and hidden eggs for 1202 amphibian species with no missing data (Figure 2a; Table S1, Supplementary Data File). Data on egg attendance by sex and direct development were taken from Furness and Capellini (2019) (Supplementary Methods). Egg attendance was defined as parents remaining full or part‐time with eggs at a fixed location. Direct development referred to eggs that hatch directly as juveniles; species where eggs hatch as tadpoles were classed as lacking direct development. Hypotheses on the relationship between egg attendance and direct development apply specifically to species with externally laid eggs. Thus, we restricted direct development to include only eggs that develop in the external environment and we did not class as direct‐developing species with brooding and viviparity. Data on terrestrial eggs came from Furness et al. (2022). Eggs were scored as terrestrial if deposited terrestrially and aquatic if deposited in water (Supplementary Methods). Data on hidden eggs were compiled for this study from 458 primary and secondary sources (Supplementary Data file). We considered eggs to be hidden if laid in a protected site, such as subterranean burrows, tree holes, cavities, nests, underneath rocks, logs, leaf litter or other structures. Exposed eggs were those laid in unprotected locations, such as floating uncovered in aquatic environments or on top of terrestrial vegetation. Species with eggs that develop on or inside the parents' body (brooding and viviparous species) were scored as not having hidden eggs, since the hypotheses on the relationship between egg attendance and hidden eggs refer specifically to species with externally laid eggs.

### Analyses

#### Overview

We used the most comprehensive time‐calibrated amphibian phylogeny built solely with molecular data without imputation of missing taxa based on taxonomy (Pyron, 2014). Reproductive ecology traits may covary with one another as well as with egg attendance. For example, direct developing eggs are often terrestrial and frequently hidden (Figure 2a; Supplementary Data file). Thus, we first used a probit model in MCMCglmm (Hadfield, 2010) with egg attendance by one sex as the response variable, and egg attendance by the other sex and all reproductive ecology traits entered simultaneously as predictors. This allowed us to account for their covariation, identify significant associations, and estimate magnitude and direction of partial effects for all predictors. We used Variance Inflation Factors (VIFs) to evaluate degree of collinearity between predictors and considered collinearity to be problematic when VIF scores were greater than 5 (Quinn & Keough, 2002). Next, we used Discrete models, which can only take two binary variables, in *BayesTraits* V.3 (Pagel & Meade, 2006) to investigate the order of evolutionary events explaining the association between each significant predictor and egg attendance.

Probit models and Discrete models were run in a Bayesian framework with Markov Chain Monte Carlo (MCMC). For all analyses, we examined the trace plots of the posterior distributions to confirm that the chains converged and exhibited adequate mixing with acceptable low levels of autocorrelation. All analyses were run in triplicate and converged on qualitatively similar solutions; here we report the results for the first chain.

#### Probit models

We modelled the probability of observing male or female egg attendance (binary response variable) as a function of all binary predictors entered together in a phylogenetic linear mixed model (de Villemereuil et al., 2013; Hadfield, 2010). We used a largely uninformative prior (normal distribution with mean of zero and variance of 10^8^) for the fixed effects and a chi‐squared prior, which approximates a cumulative uniform distribution, for the phylogeny treated as a random effect. We fixed the residual variance to 1 because this cannot be estimated in models with binary response variables (de Villemereuil et al., 2013; Hadfield, 2010). MCMC chains were run for 5 million iterations with a burnin of 100,000 and sampling every 2500. We considered a predictor to be associated with egg attendance if less than 5% of the posterior distribution of its *beta* estimate (ß) crossed zero (Px) (Capellini et al., 2015). We computed heritability (*h* ^*2*^) to quantify the importance of species' shared evolutionary history. Heritability can be interpreted in the same way as Pagel's lambda ranging from 0 (no phylogenetic effects) to 1 (strongest phylogenetic effects) (Hadfield, 2010).

Significant positive associations in probit models indicate that egg attendance is more likely to be observed when the predictor (terrestrial eggs, hidden eggs, direct development, attendance by the other sex) is present, while significant negative associations indicate the two traits are mutually exclusive as predicted by hypotheses that they are alternative adaptations to increase egg survival (Figure 1, Table 1).

#### Discrete models

We used Discrete Dependent models to reveal the order of evolutionary events for those predictors that were identified as significantly associated with egg attendance in probit models, after confirming that they were also evolutionarily associated when tested in Discrete framework (Supplementary Methods). We then examined the posterior distributions of the eight transition rates between the four possible combinations of character states in Discrete models to identify evolutionary pathways consistent with the proposed hypotheses (Figure 1, Table 1). Specifically, if reproductive ecology traits drive the evolution of egg attendance, the acquisition of egg attendance should be gained faster, that is, higher transition rate, in their presence than in their absence and/or ecological traits are gained more rapidly from the absence of both traits than egg attendance and are followed by a rapid gain of egg attendance (Figure 1a, Table 1). Alternatively, if egg attendance promotes changes in reproductive ecology, the gain of the novel reproductive ecology trait should be faster in the presence of egg attendance than in its absence and/or egg attendance should be gained faster than the reproductive ecology trait and should facilitate the rapid gain of the reproductive ecology trait (Figure 1b, Table 1). Note that transition rates estimated to be zero indicate unlikely evolutionary pathways. Finally, we can identify as evolutionary stable conditions those combinations of character states gained faster than they are lost. This can reveal if egg attendance in the presence of a given reproductive ecology trait is an evolutionarily stable condition or whether care and reproductive ecology traits are alternative adaptations. If the latter hypothesis is correct (Figure 1c), egg attendance alone or the reproductive ecology trait alone should be evolutionarily stable but the presence of both should not (Table 1).

In all discrete analyses, we scaled the branch lengths of the phylogeny by a constant (mean of 0.1), as scaling enables the algorithm to better explore parameter space when transition rates are small, hard to estimate or to search for. MCMC chains were run for 400 million iterations with a burnin of 500,000 and sampling every 200,000. We used an exponential prior with mean seeded from a uniform hyperprior ranging from 0 to 20, and Reversible Jump (RJ) in all analyses. RJ can propose models with transition rates equal to zero or to one another, helping to reduce model complexity and avoid over‐parametrisation, and are thus particularly useful for analyses where the sample size for some combination of character states is small (Currie & Meade, 2014). For each transition rate of the discrete‐dependent models, we report the mean, median, and mode of the posterior distribution, the 95% credible interval and the percentage of models that estimate that transition rate to be equal to 0.

## RESULTS

**FIGURE 3:**
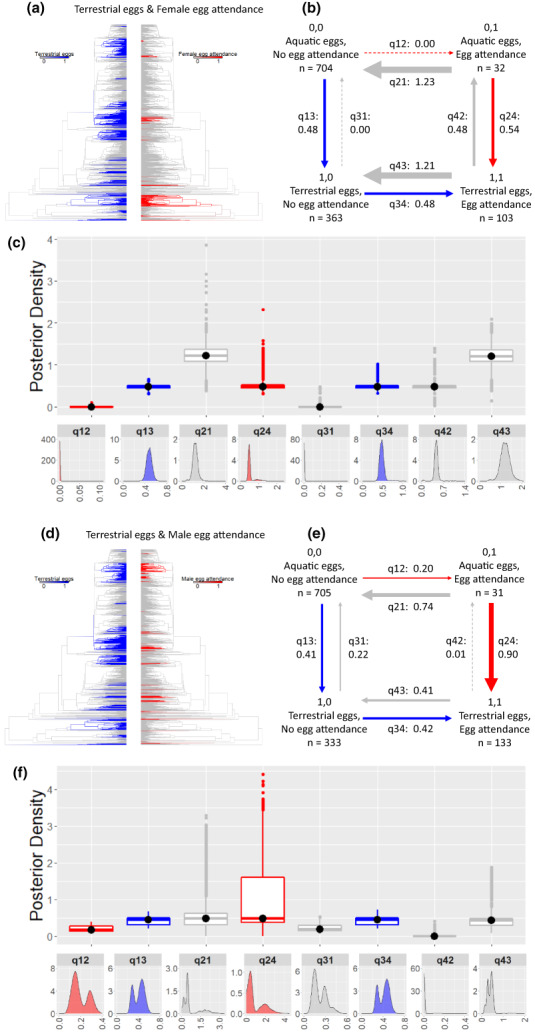
The correlated evolution of terrestrial eggs and egg attendance. Terrestrial eggs (blue) and female egg attendance (red) in (a) and male egg attendance (red) in (d) plotted on the phylogeny using stochastic character mapping with the R package phytools to visualise their evolutionary history. In (b) and (e), summary diagrams of the transition rates across the four combinations of character states from the RJ discrete‐dependent model of evolution (care first pathway highlighted by the red arrows, ecology first pathway by the blue arrows; pathways in grey indicate reversals with losses of traits). The sample sizes for each combination of character states are reported. Within each summary diagram, the arrows are scaled to reflect the magnitude of the reported mean transition rates from the posterior distribution. Arrows are dashed when a parameter is estimated to be equal to zero in over 25% of models of the posterior distribution. In (c) and (f), the posterior distributions of the transition rates from the RJ discrete‐dependent model are shown as box plots for comparison and as posterior density plots for each transition rate alone. The central black dot in the box plots indicates the median, the box the upper and lower quartiles, the vertical lines the 95% credible intervals of the posterior distributions and the filled dots beyond the lines indicate outlier estimates (Table S5 for additional transition rate summaries).

**FIGURE 4:**
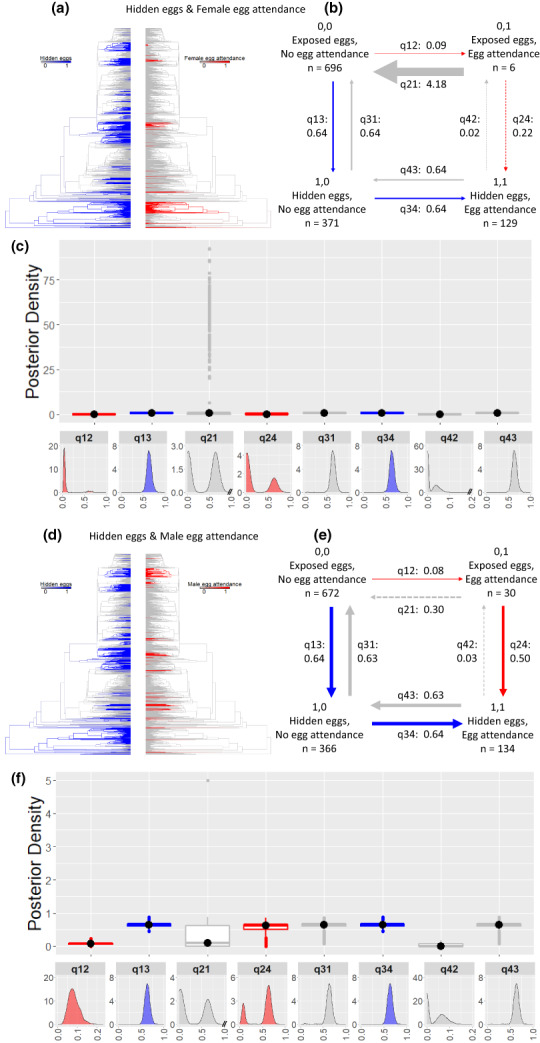
The correlated evolution of hidden eggs and egg attendance. Hidden eggs (blue) and female egg attendance (red) in (a) and male egg attendance (red) in (d) plotted on the phylogeny using stochastic character mapping with the R package phytools to visualise their evolutionary history. In (b) and (e), summary diagrams of the transition rates across the four combinations of character states from the RJ discrete‐dependent model of evolution (care first pathway highlighted by the red arrows, ecology first pathway by the blue arrows; pathways in grey indicate reversals with losses of traits). The sample sizes for each combination of character states are reported. Within each summary diagram, the arrows are scaled to reflect the magnitude of the reported mean transition rates from the posterior distribution. Arrows are dashed when a parameter is estimated to be equal to zero in over 25% of models of the posterior distribution. In (c) and (f), the posterior distributions of the transition rates from the RJ discrete‐dependent model of evolution are shown as box plots for comparison and as posterior density plots for each transition rate alone. The central black dot in the box plots indicates the median, the box the upper and lower quartiles, the vertical lines the 95% credible intervals of the posterior distributions, and the filled dots beyond the lines indicate outlier estimates (Table S6 for additional transition rate summaries).

**FIGURE 5:**
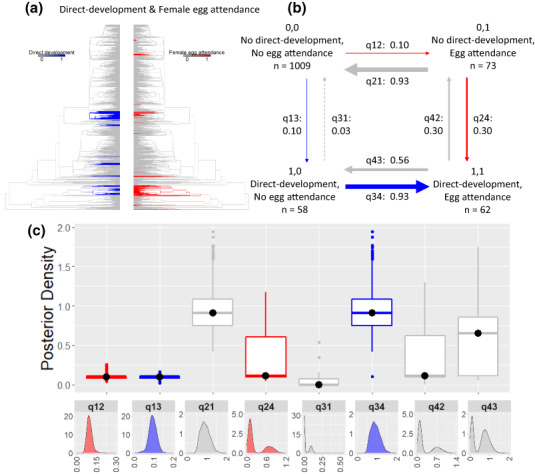
The correlated evolution of direct development and female egg attendance. In (a) direct development (blue) and female egg attendance (red) plotted on the phylogeny using stochastic character mapping with the R package phytools to visualise their evolutionary history. In (b), summary diagrams of the transition rates across the four combinations of character states from the RJ discrete‐dependent model of evolution (care first pathway highlighted by the red arrows, ecology first pathway by the blue arrows; pathways in grey highlight reversals with losses of traits). The sample sizes for each combination of character states are reported; the arrows are scaled to reflect the magnitude of the reported mean transition rates from the posterior distribution, with the mean value also indicated. Arrows are dashed when a parameter is estimated to be equal to zero in over 25% of models of the posterior distribution. In (c), the posterior distributions of the transition rates from the RJ discrete‐dependent model of evolution are shown as box plots for comparison and as posterior density plots for each transition rate alone. The central black dot in the box plots indicates the median, the box the upper and lower quartiles, the vertical lines the 95% credible intervals of the posterior distributions and the filled dots beyond the lines indicate outlier estimates (Table S7 for additional transition rate summaries).

Probit models reveal that female egg attendance is more likely to be found in species with terrestrial eggs, hidden eggs and direct development (Figure 2b; Table S2a). Similarly, male egg attendance is more likely to be observed in species with terrestrial eggs and hidden eggs but is unrelated to direct development (Figure 2c; Table S2b). Male and female egg attendance are negatively associated, consistent with our previous findings that biparental care is evolutionarily unstable and leads to the loss of care by one sex (Furness & Capellini, 2019). VIF scores indicate that there is no problematic multicollinearity between our predictors (Table S3). Discrete models confirm that female and male egg attendance are evolutionarily associated with the predictors identified by probit models (Figures 3a,d, 4a,d, 5a; Table S4).

Discrete Dependent models indicate that female egg attendance is very unlikely to evolve in aquatic habitats (99% of the models estimate q_12_ to be 0) but is gained when terrestrial eggs are present (q_34_ > q_12_) (Figures 3b,c, Table S5a). From the absence of both traits, terrestrial eggs are gained at a faster rate than female egg attendance (q_13_ > q_12_) (Figures 3b,c, Table S5a). These results indicate that female egg attendance follows the evolution of terrestrial egg development. Moreover, female attendance of aquatic eggs evolves through the loss of terrestrial egg development (q_42_; Figures 3b,c; Table S5a). Female egg attendance is however evolutionarily labile and rapidly lost with both aquatic and terrestrial eggs (transition rates q_21_ and q_43_ are the highest; Figures 3b,c; Table S5a).

Although male egg attendance and terrestrial eggs are evolutionarily correlated (Figures 2c, 3d; Tables S2b, S4, S5b), the transition rates for the acquisition of terrestrial eggs when male egg attendance is present versus absent (q_13_; q_24_), and of egg attendance when eggs are aquatic or terrestrial (q_12_, q_34_), overlap substantially, indicating they are of similar magnitude (Figures 3e,f; Table S5b). Thus, no clear evolutionary pathway through which these traits have become associated can be identified in males. As in females, male egg attendance is lost in both aquatic and terrestrial habitats (q_21_, q_43_) but, contrary to females, attendance of terrestrial eggs is unlikely to favour a reversal to attendance of aquatic eggs (Figures 3e,f; Table S5b). Instead, male attendance of aquatic eggs is more likely to evolve through the acquisition of care in aquatic habitats (q_12_ > q_42_; Figures 3e,f; Table S5b).

Male and female attendance of hidden eggs exhibit similar evolutionary trajectories: egg attendance evolves faster with hidden than exposed eggs (q_34_ > q_12_) and at a slower rate than hidden eggs from the absence of both traits (q_13_ > q_12_) (Figures 4b,c,e,f; Table S6). Attending exposed eggs is an evolutionarily unstable condition which leads to the rapid loss of egg attendance, particularly in females or, especially in males, to laying eggs in hidden sites (Figures 4b,c,e,f; Tables S6). Finally, attendance in both sexes can be lost as quickly as gained when the eggs are hidden (q_34_ equal to q_43_) (Figures 4b,c,e,f; Tables S6). Thus, the most likely pathway from exposed eggs without egg attendance to hidden eggs with egg attendance is through an intermediate state of hidden eggs without attendance.

Finally, while direct development and female egg attendance evolve at a similar rate from a condition where both are absent (q_12_ similar to q_13_), egg attendance is gained nearly an order of magnitude faster with direct‐developing eggs than with eggs hatching as tadpoles (q_34_ > q_12_). In contrast, the posterior distribution of q_13_ and q_24_ overlap substantially indicating that direct development evolves at similar rates with and without female egg attendance (Figures 5b,c, Table S7). Once evolved, female egg attendance can be quickly lost regardless of whether eggs hatch directly as juveniles or tadpoles (Figure 5b, Table S7). These results indicate that direct development precedes the evolution of female egg attendance.

## DISCUSSION

Whether reproductive ecology promotes the evolution of parental care is an open question. Here we tested hypotheses suggesting that reproductive ecology facilitates the origin of amphibian egg attendance, and counter‐hypotheses proposing either the opposite direction of causation, or that care and reproductive ecology represent alternative adaptations to increase egg survival. Our study reveals that terrestrial egg laying facilitates the origin of female egg attendance but could not identify whether male egg attendance follows or precedes the transition to terrestrial egg development. Hidden eggs promote the evolution of both male and female egg attendance, while direct development facilitates only the acquisition of female egg attendance. Thus, we find limited evidence for hypotheses proposing reversed causation and no evidence that changes in reproductive ecology are alternative adaptations to providing care in amphibians.

While we find strong evidence that both male and female egg attendance are evolutionarily associated with terrestrial egg deposition, our analysis identified a clear evolutionary pathway for females only. Specifically, female egg attendance follows the shift to terrestrial egg development as predicted by the hypothesis that terrestrial reproduction drives the evolution of parental care (Crump, 1995; Gomez‐Mestre et al., 2012; McDiarmid, 1978; Salthe & Mecham, 1974). Although terrestrial egg development requires adaptations against desiccation (McDiarmid, 1978; Seymour, 1999; Wells, 2007), the survival of anamniotic eggs on land is probably not as difficult as assumed, particularly in environments with high humidity (Gomez‐Mestre et al., 2012). Egg deposition in terrestrial habitats has evolved in several fish families (Martin & Carter, 2013), is widespread in insects (Church et al., 2019) and, like for amphibians, is believed to be an adaptation to reduce egg predation (Martin & Carter, 2013). Because amphibian terrestrial eggs are large and laid in small clutches (Furness et al., 2022; Gomez‐Mestre et al., 2012), their high fitness value is a likely driving force promoting the evolution of female egg attendance since female fitness is constrained by the number of eggs produced (Gomez‐Mestre et al., 2012; Smith & Fretwell, 1974).

Although our analysis shows that male egg attendance and terrestrial eggs have evolved together and are not alternative adaptations, the pathway through which they become evolutionarily associated remains unclear. Other ecological and social conditions may need to be considered to identify the evolutionary pathway in males. For example, eggs may suffer different levels of predation risk or oxygen limitation across different types of aquatic or terrestrial habitats, and male egg attendance may evolve in more specific habitats. Social factors, such as male territoriality, may also facilitate the origin of male care in amphibians and other taxa (Gross & Sargent, 1985; Trivers, 1972; Vági et al., 2020; Williams, 1975). Specifically, if males defend territories in which females lay clutches, the cost of attendance may be lowered as caring males may not miss out on additional mating opportunities (Kvarnemo, 2006). Further, if attending eggs signal male quality, parental care may increase males' chances to attract further mates (Forsgren et al., 1996).

Consistent with the hypothesis that hiding eggs may reduce predation risk to eggs and parents and drive the evolution of parental care (Crump, 1995), we find that hiding eggs evolve first and promote the acquisition of egg attendance in both sexes. These results suggest that caring parents undertake other important tasks beyond protection against predators that enhance egg survival and select for egg attendance, like hydrating hidden eggs and maintaining water balance (Taigen et al., 1984). Our analysis reveals that attendance of exposed eggs evolves slowly and is evolutionarily unstable in both sexes but, while it promotes the rapid acquisition of hidden eggs in males, egg attendance is more likely lost in females. Few extant amphibian species thus retain the peculiar combination of attendance of exposed eggs, likely due to the high predation risk for caring parents. To reduce this risk, some glassfrog males attend to exposed terrestrial eggs at night but remain concealed during the day (McDiarmid, 1978) when visually oriented predators are most active.

We proposed that direct‐developing eggs could facilitate the origin of egg attendance particularly in females. These eggs are large, laid in small clutches (Furness et al., 2022) and have prolonged developmental periods that expose them to mortality risks for longer. The high fitness value of large eggs should promote female attendance (Trivers, 1972) because, unlike for males, female fitness is limited by fecundity. We find strong support for this hypothesis as female egg attendance is gained at much faster rates with direct‐developing eggs than in eggs hatching as tadpoles, suggesting that direct development selects for female egg attendance. Future studies should investigate whether terrestrial, unattended direct‐developing eggs exhibit adaptations that increase their survival, such as chemical resistance to fungus, desiccation‐resistant outer layers or thick jelly layers, as in species lacking direct development (Delia et al., 2020; Green, 1999; Seymour, 1999).

Research has revealed the costs and benefits of parental care in many species but understanding why care has evolved in some taxa but not others have remained elusive (Royle et al., 2012). Here we show that shifts in reproductive ecology—terrestrial egg development, hiding eggs, direct development—promote the origin of the simplest form of parental care—egg attendance, particularly in females. Direct development and terrestrial egg development in amphibians also select for small clutches of large eggs (Furness et al., 2022). Few large eggs represent a major investment with high fitness return, particularly for females (Smith & Fretwell, 1974). Combined, this suggests that the evolution of egg attendance is favoured in females when few large eggs evolve as a result of changes in reproductive ecology (e.g. terrestrial eggs; direct development); in both sexes when the costs of caring can be reduced by hiding the eggs; and in males that already defend territories (Gross & Sargent, 1985; McDiarmid, 1978). Importantly, once evolved, male and female egg attendance promote further parental investment in larger eggs (Furness et al., 2022) and care at the tadpole and juvenile stage (Furness & Capellini, 2019), as expected by theoretical models (Trivers, 1972). Multiple functions of egg attendance that increase egg survivorship have already been documented in few well‐studied species. Once detailed information becomes available for many amphibian species, future studies could investigate if some functions promote the origin of egg attendance while others have been acquired later and help maintain this simple care behaviour.

Drivers for the origin of parental care do not necessarily select for its persistence over evolutionary time (Royle et al., 2016). Indeed, egg attendance in amphibians is evolutionarily labile since it is gained as quickly as it is lost (Furness & Capellini, 2019) and here we show that the reproductive ecology drivers promoting its origin do not help retain it. Interactions between parents and offspring are likely to determine whether parental care is maintained, increased through the acquisition of further parental investment, or lost (Furness & Capellini, 2019). The complex dynamics of cooperation and conflict between family members likely contribute to this outcome. For example, care forms in which offspring have the opportunity to manipulate parental behaviour, through chemical communication in viviparous matrotrophy and brooding or through begging, may limit the opportunity of losing care compared to care forms, such as egg attendance, that allow parents greater control over the amount of care provided (Furness & Capellini, 2019). Similarly, evolutionary changes in offspring that make them more dependent on parental care are likely to prevent the loss of care (Jarrett et al., 2018).

In conclusion, egg attendance is the most common form of care in the animal kingdom (Balshine, 2012; Smiseth et al., 2012), increases offspring survival (García‐Hernández & Machado, 2017; Klug et al., 2005; Ospina‐L et al., 2020; Pike et al., 2016), selects for larger eggs (Furness et al., 2022) and may trigger the acquisition of care at later stages of offspring development (Furness & Capellini, 2019). This study reveals that female egg attendance in particular is more likely to evolve following changes in reproductive ecology that likely increase egg survival, select for small clutches of large eggs, and/or expose eggs to new environmental challenges. Reproductive ecology traits similar to those studied here are common in other taxa such as insects, fish and crustaceans, in which diverse forms of parental care have been documented. Thus, we expect that, if reproductive ecology traits decrease the costs of caring and/or increase the fitness value of a clutch, they may have facilitated the origin of parental care, particularly simpler forms like egg attendance, in these taxa as they do in amphibians.

## AUTHOR CONTRIBUTIONS

IC and AIF designed the study, AIF assembled the data set and conducted analyses with guidance from IC, and IC and AIF wrote the manuscript.

### PEER REVIEW

The peer review history for this article is available at https://publons.com/publon/10.1111/ele.14109.

## Supporting information


Table S1
Table S2
Table S3
Table S4
Table S5
Table S6
Table S7

## Data Availability

The data set, data set references and phylogenetic tree are available on figshare (10.6084/m9.figshare.20658138).
