# Supplementary material for: The reproductive ecology drivers of egg attendance in amphibians
Source: Ecol Lett. 2022 Oct 1;25(11):2500–12. doi: 10.1111/ele.14109 (PMC9827844; doi:10.1111/ele.14109)
Supplement: Supplementary file 1 — Table S1 Table S2 Table S3 Table S4 Table S5 Table S6 Table S7 [file ELE-25-2500-s001.docx]

**Supplementary** **Information**

**Supplementary Methods**

Data set

Here we give further details regarding the scoring of egg attendance by sex, direct development, terrestrial eggs, and hidden eggs.

*Egg attendance*

Data on egg attendance by sex was taken from Furness and Capellini (2019). Egg attendance was identified when parent(s) remain with the eggs, full or part-time, at a fixed location, typically from oviposition until hatching. Egg attending parents defend eggs against predators (Delia et al. 2017), rotate eggs to prevent fungus formation or remove unhealthy eggs (Green 1999), aerate aquatic eggs (Takahashi et al. 2017), and hydrate terrestrial eggs (Taigen et al. 1984). Such functions increase egg survival but are frequently costly as attending parents may be susceptible to predation or less likely to feed (Crump 1995, Wells 2007, Delia et al. 2017). We excluded from our definition of egg attendance the few cases in which attendance lasted less than a few hours immediately following oviposition after which the eggs were permanently abandoned.

*Direct development*

Direct development data came from Furness et al. (2019), and referred to eggs that hatch directly as juveniles, without a tadpole stage. Species where eggs hatch as tadpoles were classed as lacking direct development. Thus, all species were scored for presence or absence of direct development, regardless of oviposition location or whether or not the eggs were attended. Here, brooding and viviparous species, in which the eggs develop on or inside the parents’ body, were scored as not having direct development, since the hypotheses on the relationship between egg attendance and direct development refer specifically to eggs laid in the external environment.

*Terrestrial eggs*

Data on terrestrial versus aquatic egg deposition was taken from Furness et al. (2022). Eggs were scored as aquatic when they developed in water irrespective of the size or location of the waterbody. Included in this category were eggs deposited in rivers, lakes, small and large pools including in water-filled phytotelmata, in foam nests laid on the water surface, foam nests in excavated basins filled with water, foam nests on the water’s edge, and a few brooding and viviparous species that are fully aquatic (family Pipidae and Typhlonecitade). Eggs were scored as terrestrial when they developed away from water, either on the ground (for example amongst leaf litter, in burrows, holes, or cavities, and inside terrestrial foam nests), arboreally (for example attached to vegetation and leaves, on the sides of tree holes), or on or inside the body of terrestrial brooding and viviparous species.

*Hidden eggs*

Egg deposition location is fairly consistent within a species from descriptions in a large number of sources including papers, books, and online professional databases (Supplementary Data file). We classified as hidden eggs those that are laid in protected sites such as subterranean burrows, tree holes, cavities, nests, underneath rocks, logs, leaf litter, or other structures. Some species with aquatic egg deposition have hidden eggs such as eggs placed under rocks on the stream bottom, in underwater burrows, or individually wrapped in aquatic vegetation as in some newts. Exposed eggs were those laid in unprotected locations. Exposed terrestrial eggs are laid on top of and attached to terrestrial vegetation (i.e. leaves), and exposed aquatic eggs are laid uncovered, and often floating, in waterbodies such as rivers, lakes, ponds, and pools. Hypotheses on the relationship between egg attendance and hidden eggs apply specifically to species with externally laid eggs. Thus, we did not class brooding and viviparous species as having hidden eggs.

Analysis

To confirm that egg attendance and the significant predictions in probit models were evolutionarily associated, we compared the fit to the data of two alternative models in Discrete: a Discrete Independent model, in which the two traits evolve independently of each other, and a Discrete Dependent model in which the evolution of one trait is contingent on that of the other (Pagel 1994; Pagel & Meade 2006). We estimated the marginal likelihoods of the Independent and Dependent models in *BayesTraits* using a stepping stone sampler (Pagel *et al.* 2004; Xie *et al.* 2011) set to 200 stones and 200,000 iterations per stone. We then computed Bayes Factors (BF) as twice the difference in the logarithm of the models’ marginal likelihoods to identify which model fit the data better. We considered evidence that the Dependent model fit better than the alternative Independent model when BF were greater than 2, strong evidence when BF were greater than 5, and very strong evidence when BF were 10 or above (Pagel & Meade 2006).

**Supplementary References**

Crump, M.L. (1995). Parental care. In: *Amphibian biology* (ed. Heatwole, H). Surrey Beatty & Sons Chipping Norton, N.S.W., pp. 518-567.

Delia, J., Bravo‐Valencia, L. & Warkentin, K.M. (2017). Patterns of parental care in Neotropical glassfrogs: fieldwork alters hypotheses of sex‐role evolution. *Journal of Evolutionary Biology*, 30, 898-914.

Furness, A.I. & Capellini, I. (2019). The evolution of parental care diversity in amphibians. *Nature Communications*, 10, 1-12.

Furness, A.I., Venditti, C. & Capellini, I. (2022). Terrestrial reproduction and parental care drive rapid evolution in the tradeoff between offspring size and number. *PLOS Biology*, 20, e3001495.

Green, A.J. (1999). Implications of pathogenic fungi for life-history evolution in amphibians. *Functional Ecology*, 13, 573-575.

Pagel, M. (1994). Detecting correlated evolution on phylogenies: a general method for the comparative analysis of discrete characters. *Proceedings of the Royal Society B*, 255, 37-45.

Pagel, M. & Meade, A. (2006). Bayesian analysis of correlated evolution of discrete characters by reversible-jump Markov chain Monte Carlo. *The American Naturalist*, 167, 808-825.

Taigen, T.L., Pough, F.H. & Stewart, M.M. (1984). Water balance of terrestrial anuran (*Eleutherodactylus coqui*) eggs: importance of parental care. *Ecology*, 65, 248-255.

Takahashi, M.K., Okada, S. & Fukuda, Y. (2017). From embryos to larvae: seven‐month‐long paternal care by male Japanese giant salamander. *Journal of Zoology*, 302, 24-31.

Wells, K.D. (2007). *The ecology and behavior of Amphibians*. University of Chicago Press, Chicago.

Xie, W., Lewis, P.O., Fan, Y., Kuo, L. & Chen, M.-H. (2011). Improving marginal likelihood estimation for Bayesian phylogenetic model selection. *Systematic Biology*, 60, 150-160.

**Table S1. Sample sizes for categorical, binary, independent variables considered as predictors**

**of male and female egg attendance.** Only 16 species have both male and female egg attendance (i.e. are biparental). The total sample size of our data set is 1202 species, with no missing data.

| **Variable** | **Absent (0)** | **Present (1)** |
| --- | --- | --- |
| Female egg attendance | 1067 | 135 |
| Male egg attendance | 1038 | 164 |
| Terrestrial eggs | 736 | 466 |
| Direct development | 1082 | 120 |
| Hidden eggs | 702 | 500 |

**Table S2. Results of analyses with probit models for the evolution of sex-specific egg attendance and all ecological predictors.** We report the mean, 95% credible intervals, and the percentage of the posterior distribution that crosses 0 (Px) for the predictors (see also Figure 2). We consider evidence of significance Px < 0.05.

| 1. **Female egg attendance and ecological predictors** | | | | |
| --- | --- | --- | --- | --- |
| **Parameter** | **mean** | **l-95% CI** | **u-95% CI** | **P_x_** |
| Intercept | -4.495 | -7.419 | -1.670 | 0.000 |
| Terrestrial eggs | 1.331 | 0.414 | 2.331 | 0.004 |
| Direct development | 1.451 | 0.182 | 2.696 | 0.011 |
| Hidden eggs | 2.103 | 1.081 | 3.182 | 0.000 |
| Male egg attendance | -0.993 | -1.902 | -0.148 | 0.008 |
| Heritability | 0.831 | 0.713 | 0.906 | - |

| 1. **Male egg attendance and ecological predictors** | | | | |
| --- | --- | --- | --- | --- |
| **Parameter** | **mean** | **l-95% CI** | **u-95% CI** | **P_x_** |
| Intercept | -7.980 | -12.212 | -3.698 | 0.000 |
| Terrestrial eggs | 2.282 | 1.096 | 3.482 | 0.000 |
| Direct development | -0.700 | -2.396 | 1.141 | 0.228 |
| Hidden eggs | 3.550 | 2.354 | 4.936 | 0.000 |
| Female egg attendance | -1.520 | -2.705 | -0.415 | 0.004 |
| Heritability | 0.924 | 0.884 | 0.950 | - |

**Table S3. Variance inflation factors (VIF) from the probit models for female egg attendance (a) and male egg attendance (b).** VIF scores are considered indicative of potentially problematic multicollinearity when equal or greater than 5.

| 1. **Female egg attendance** | |
| --- | --- |
| **Parameter** | **VIF** |
| Terrestrial eggs | 1.46 |
| Direct development | 1.29 |
| Hidden eggs | 1.39 |
| Male egg attendance | 1.19 |

| 1. **Male egg attendance** | |
| --- | --- |
| **Parameter** | **VIF** |
| Terrestrial eggs | 1.38 |
| Direct development | 1.42 |
| Hidden eggs | 1.42 |
| Female egg attendance | 1.32 |

**Table S4. Correlated evolution of sex-specific egg attendance and ecological predictors.** Comparison between Discrete Dependent and Independent models using BayesTraits. Each line depicts the results of separate analyses with two variables. The first two columns report the two traits tested: sex-specific egg attendance as a binary absence/presence trait and the predictor, terrestrial eggs (absent/present), hidden eggs (absent/present), and direct development (absent/present). We report the Bayes Factor (BF) as a measure of support for the Dependent (correlated evolution) model against the Independent model. BF greater than 2 indicate support for the dependent model, greater than 5 strong support, and greater than 10 very strong support; negative BF indicate greater support for the Independent model.

| **Care** | **Reproductive ecology** | **BF** | **Supported model** |
| --- | --- | --- | --- |
| Female egg attendance | Terrestrial eggs | 30.7 | Dependent |
| Male egg attendance | Terrestrial eggs | 12.0 | Dependent |
| Female egg attendance | Hidden eggs | 23.0 | Dependent |
| Male egg attendance | Hidden eggs | 23.5 | Dependent |
| Female egg attendance | Direct development | 14.8 | Dependent |
| Male egg attendance | Direct development | -5.4 | Independent |

**Table S5. Correlated evolution of egg attendance and terrestrial eggs from *BayesTraits* RJ Discrete Dependent models.** In (a) female egg attendance and terrestrial eggs; in (b) male egg attendance and terrestrial eggs. The columns report the magnitude of the transition rates between combinations of character states (see Figure 3), the mean, median and mode of the posterior distributions, the 95% highest posterior density (HPD) interval, and the percentage of models in the posterior in which a given parameter is estimated as equal to 0 (% zero).

| 1. **Female egg attendance and terrestrial eggs** | | | | | |
| --- | --- | --- | --- | --- | --- |
| **Transition rate** | **Mean** | **Median** | **Mode** | **95% HPD** | **% Zero** |
| q12 | 0.00 | 0.000 | 0.000 | [0, 0] | 99.3 |
| q13 | 0.48 | 0.477 | 0.486 | [0.3884, 0.5737] | 0 |
| q21 | 1.23 | 1.219 | 1.133 | [0.7777, 1.6844] | 0 |
| q24 | 0.54 | 0.486 | 0.486 | [0.3618, 1.0266] | 0 |
| q31 | 0.00 | 0.000 | 0.000 | [0, 0] | 98.6 |
| q34 | 0.48 | 0.481 | 0.486 | [0.3819, 0.5850] | 0 |
| q42 | 0.48 | 0.478 | 0.486 | [0.3781, 0.5862] | 0 |
| q43 | 1.21 | 1.209 | 1.114 | [0.7765, 1.6644] | 0 |

| 1. **Male egg attendance and terrestrial eggs** | | | | | |
| --- | --- | --- | --- | --- | --- |
| **Transition rate** | **Mean** | **Median** | **Mode** | **95% HPD** | **% Zero** |
| q12 | 0.20 | 0.173 | 0.151 | [0.0908, 0.3316] | 0.0 |
| q13 | 0.41 | 0.436 | 0.460 | [0.2493, 0.5646] | 0.0 |
| q21 | 0.74 | 0.471 | 0.477 | [0, 2.2427] | 4.7 |
| q24 | 0.90 | 0.476 | 0.466 | [0, 2.6858] | 9.4 |
| q31 | 0.22 | 0.188 | 0.148 | [0.0910, 0.4165] | 0.0 |
| q34 | 0.42 | 0.436 | 0.460 | [0.2493, 0.5654] | 0.0 |
| q42 | 0.01 | 0.000 | 0.000 | [0, 0] | 95.1 |
| q43 | 0.41 | 0.420 | 0.464 | [0.1339, 0.5794] | 0.0 |

**Table S6. Correlated evolution of egg attendance and hidden eggs from *BayesTraits* RJ Discrete Dependent models.** In (a) female egg attendance and hidden eggs; in (b) male egg attendance and hidden eggs. The columns report the magnitude of the transition rates between combinations of character states (see Figure 4), the mean, median and mode of the posterior distributions, the 95% highest posterior density (HPD) interval, and the percentage of models in the posterior in which a given parameter is estimated as equal to 0 (% zero).

| 1. **Female egg attendance and hidden eggs** | | | | | |
| --- | --- | --- | --- | --- | --- |
| **Transition rate** | **Mean** | **Median** | **Mode** | **95% HPD** | **% Zero** |
| q12 | 0.09 | 0.049 | 0.042 | [0.0105, 0.5945] | 0.0 |
| q13 | 0.64 | 0.639 | 0.632 | [0.5316, 0.7549] | 0.0 |
| q21 | 4.18 | 0.595 | 0.622 | [0, 42.3124] | 20.8 |
| q24 | 0.22 | 0.042 | 0.000 | [0, 0.6959] | 37.9 |
| q31 | 0.64 | 0.637 | 0.623 | [0.5239, 0.7551] | 0.0 |
| q34 | 0.64 | 0.638 | 0.632 | [0.5310, 0.7551] | 0.0 |
| q42 | 0.02 | 0.000 | 0.000 | [0, 0.0650] | 61.9 |
| q43 | 0.64 | 0.639 | 0.632 | [0.5310, 0.7551] | 0.0 |

| 1. **Male egg attendance and hidden eggs** | | | | | |
| --- | --- | --- | --- | --- | --- |
| **Transition rate** | **Mean** | **Median** | **Mode** | **95% HPD** | **% Zero** |
| q12 | 0.08 | 0.079 | 0.084 | [0.0299, 0.1340] | 0.1 |
| q13 | 0.64 | 0.637 | 0.634 | [0.5326, 0.7533] | 0.0 |
| q21 | 0.30 | 0.304 | 0.000 | [0, 0.7066] | 29.4 |
| q24 | 0.50 | 0.613 | 0.633 | [0.0413, 0.7296] | 0.3 |
| q31 | 0.63 | 0.635 | 0.634 | [0.5206, 0.7555] | 0.0 |
| q34 | 0.64 | 0.637 | 0.637 | [0.5331, 0.7531] | 0.0 |
| q42 | 0.03 | 0.000 | 0.000 | [0, 0.1027] | 52.0 |
| q43 | 0.63 | 0.636 | 0.634 | [0.5277, 0.7552] | 0.0 |

**Table S7. Correlated evolution of female egg attendance and direct development from *BayesTraits* RJ Discrete Dependent models.** The columns report the magnitude of the transition rates between combinations of character states (see Figure 5), the effective sample size (ESS), the mean, median and mode of the posterior distributions, the 95% highest posterior density (HPD) interval, and the percentage of models in the posterior in which a given parameter is estimated as equal to 0 (% zero).

| **Female egg attendance and direct development** | | | | | |
| --- | --- | --- | --- | --- | --- |
| **Transition rate** | **Mean** | **Median** | **Mode** | **95% HPD** | **% Zero** |
| q12 | 0.10 | 0.097 | 0.096 | [0.0592, 0.1398] | 0.0 |
| q13 | 0.10 | 0.095 | 0.096 | [0.0553, 0.1363] | 0.0 |
| q21 | 0.93 | 0.909 | 0.843 | [0.5359, 1.3877] | 0.0 |
| q24 | 0.30 | 0.114 | 0.104 | [0.0611, 0.8454] | 0.0 |
| q31 | 0.03 | 0.000 | 0.000 | [0, 0.1145] | 69.8 |
| q34 | 0.93 | 0.909 | 0.843 | [0.5359, 1.3877] | 0.0 |
| q42 | 0.30 | 0.113 | 0.099 | [0.0618, 0.9336] | 0.5 |
| q43 | 0.56 | 0.651 | 0.790 | [0.0650, 1.1477] | 0.0 |
